# The Role of Hydroxychloroquine in the Management of Rheumatic Disorders: A Comprehensive Review

**DOI:** 10.1111/bcpt.70082

**Published:** 2025-07-22

**Authors:** Ilker Ates, Hilal Sahin, Lalu Muhammad Irham, Serkan Yilmaz, Sinan Suzen

**Affiliations:** ^1^ Faculty of Pharmacy, Department of Toxicology Ankara University Ankara Türkiye; ^2^ Faculty of Medicine, Department of Medical Biology and Genetic Atlas University Istanbul Türkiye; ^3^ Faculty of Pharmacy Universitas Ahmad Dahlan Yogyakarta Indonesia; ^4^ Faculty of Pharmacy Silpakorn University Nakhon Pathom Thailand; ^5^ Faculty of Nursing, Department of Midwifery Ankara University Ankara Türkiye

**Keywords:** effect mechanisms, hydroxychloroquine, organ toxicity, rheumatic disorders

## Abstract

A drug preferred for its antimalarial effect called hydroxychloroquine (HCQ) has long been used to manage and avoid malaria. Nevertheless, its exact mode of action is still unknown. HCQ works through a variety of strategies to influence distinct molecular and cellular pathways. Additionally, HCQ has been demonstrated to be an effective treatment for rheumatic conditions such as primary Sjögren's syndrome, rheumatoid arthritis, antiphospholipid syndrome and systemic lupus erythematosus. Despite being widely regarded as safe, HCQ has been known to cause adverse responses; thus, doctors should closely evaluate rheumatism patients before taking these medications. The current study aims to emphasize the potential side effects of treatment while supporting the clinical usage of HCQ for autoimmune disorders.

## Summary

- Hydroxychloroquine (HCQ) is a drug commonly used to prevent and treat malaria, though its exact mechanism is not fully understood.
- It also helps manage autoimmune diseases like rheumatoid arthritis, lupus and others.
- While generally considered safe, HCQ can cause side effects, so careful evaluation is needed before use in patients with rheumatic conditions.
- This study highlights both the benefits and potential risks of using HCQ for autoimmune disorders.

## 1 Introduction

In the early decade of the last century, the preferred medication for treating malaria was the antimalarial agent chloroquine [1]. Among other rheumatic conditions, its derivative hydroxychloroquine (HCQ) is frequently used to treat immune‐mediated antiphospholipid syndrome (APS), primary Sjögren's syndrome (pSS), rheumatoid arthritis (RA) and systemic lupus erythematosus (SLE) [2, 3, 4, 5]. As an immune‐modulating medication, HCQ has been used to bring autoimmune disorders into remission. Additionally, it lessens side effects from synthetic disease‐modifying antirheumatic medications (DMARDs) and overdose corticosteroids. HCQ is therefore regarded as a steroid‐sparing drug [6]. In general, HCQ's toleration is adequate. Although some safety data have been gathered since its clinical use as a DMARD and antimalarial medication, adverse events are still possible. Clinicians can better treat HCQ‐related adverse effects by considering both its advantages and disadvantages and keeping these possible bad consequences in consideration.

## 2 HCQ's Pharmacological Properties

**FIGURE 1:**
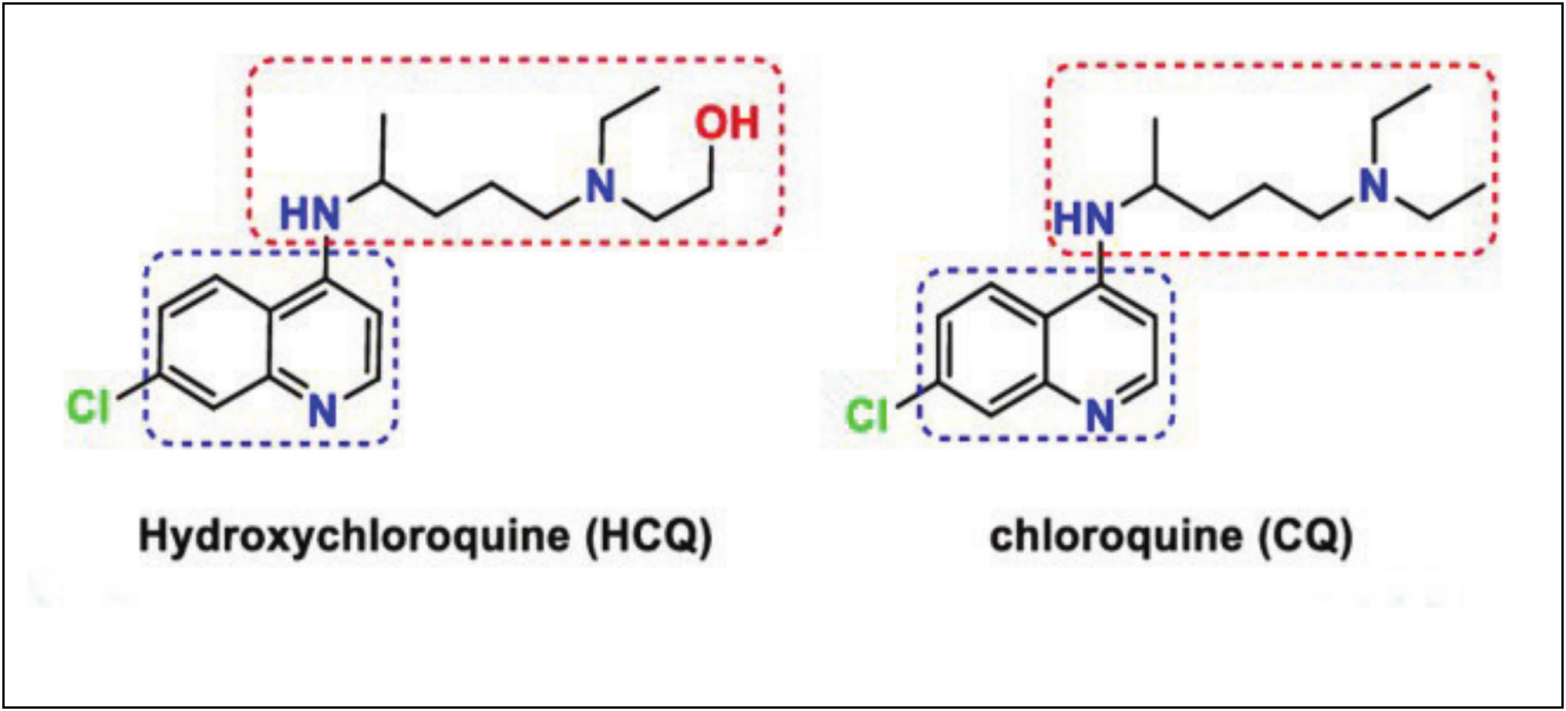
Chemical structures of chloroquine and hydroxychloroquine [8].

With anti‐inflammatory and antimalarial properties, HCQ is a hydroxylated derivative of chloroquine (Figure 1). Protonated HCQ enters the cell, and its quantity correlates negatively with pH. Consequently, it builds up in acidic organelles, such as Golgi vesicles, lysosomes and endosomes, raising their pH [7]. When taken as HCQ sulphate, it exhibits outstanding oral absorption and bioavailability. With a long mean residence period of 1300 h and an enormous distribution volume, HCQ is a poor basis. About 62% of drug metabolites have unaltered renal clearance, while 21% have changed renal clearance. Cytochrome P450 metabolizes HCQ as it travels via the liver. Following metabolic processes, 16% of HCQ is changed into desethyl HCQ and 18% into desethyl chloroquine. The ultimate half life is 45 ± 15 days [7].

## 3 Action Mechanisms of HCQ

**FIGURE 2:**
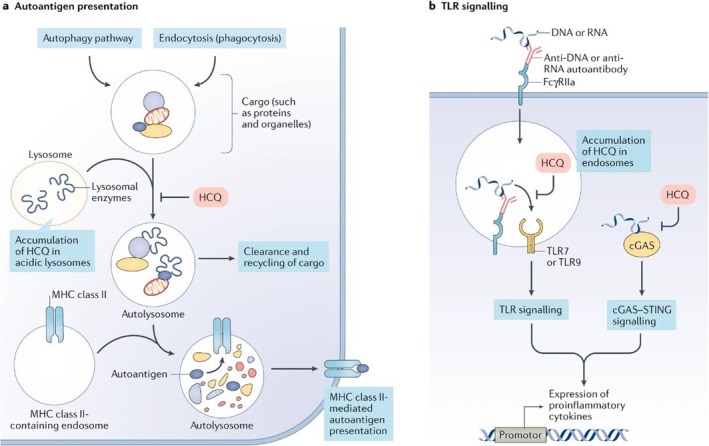
Possible molecular processes via which hydroxychloroquine contributes to autoimmunity [9]. (a) Across a pH gradient, HCQ penetrates and builds up in lysosomes. By raising the pH to stop lysosomal enzyme function, hydroxychloroquine stops cargo from being broken down in autolysosomes that are derived either internally (through the autophagy route) or externally (via endocytosis or phagocytosis). MHC class II‐mediated autoantigen presentation can be avoided by inhibiting lysosomal action. (b) Hydroxychloroquine can also attach to the minor gap of DNA with double strands and build‐up in endosomes. By changing the pH of endosomes, which are important in TLR processing, and/or blocking TLR7 and TLR9 from attaching their ligands (DNA and RNA, respectively), this medication can suppress Toll‐like receptor (TLR) signalling. By disrupting its ability to bind to cytosolic DNA, hydroxychloroquine may additionally diminish the function of the nucleic acid biosensor cyclic GMP‐AMP (cGAMP) synthase (cGAS). The generation of proinflammatory cytokines, such as type I interferons, can be decreased by hydroxychloroquine through blocking TLR signalling and cGAS–stimulator of interferon genes (STING) signalling.

**FIGURE 3:**
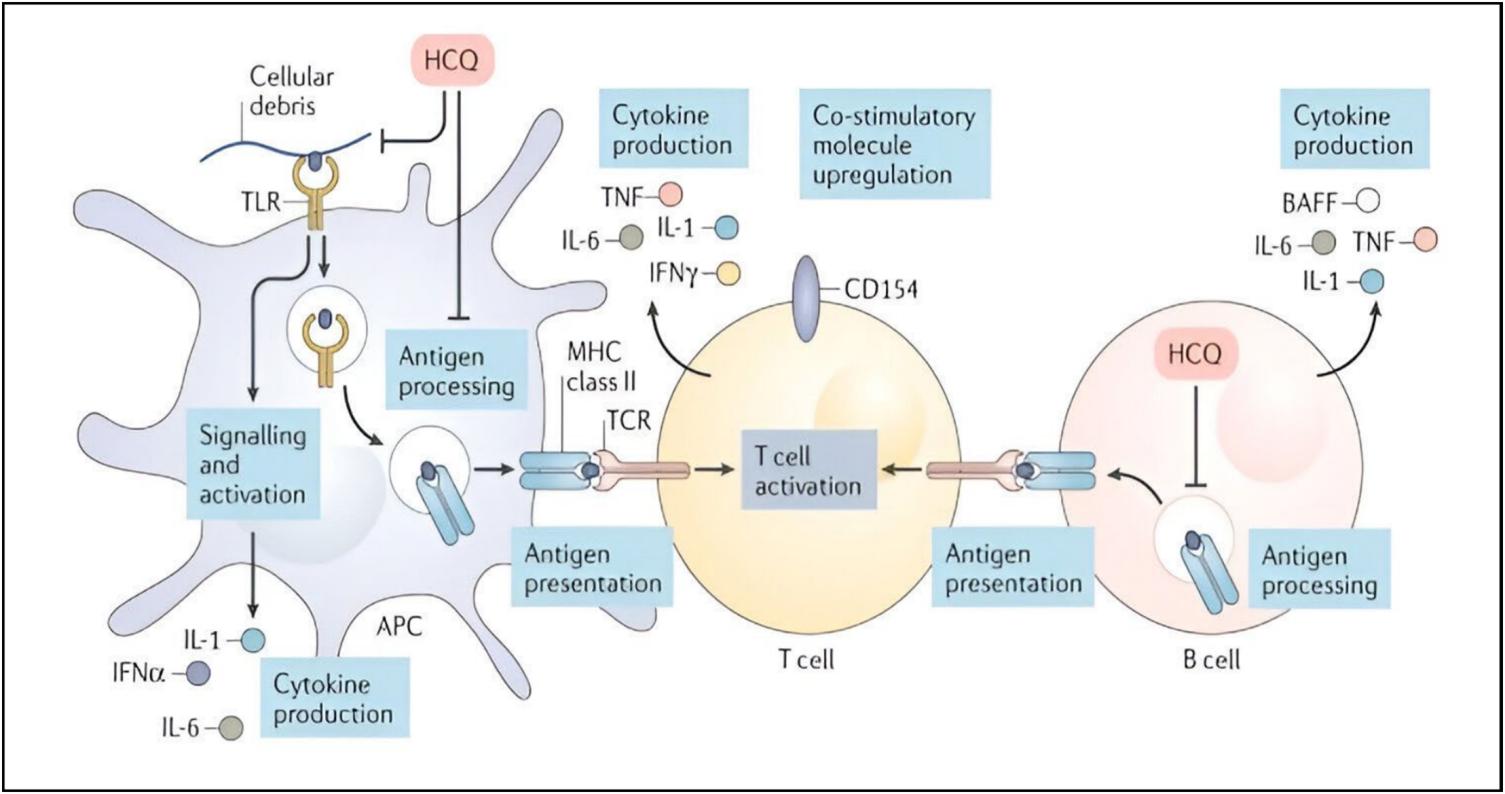
By blocking a number of innate and adaptive immunological pathways, HCQ can disrupt the activation of immunity at different cell stages. In plasmacytoid dendritic cells (pDCs) along with other immunized antigen‐presenting cells (APCs), such as monocytes, macrophages and B cells, cellular debris may stimulate the Toll‐like receptor 7 (TLR7) and TLR7 pathways of signalling throughout autoimmune diseases. This can stimulate various kinds of cells and cause the release of different proinflammatory cytokines. Via lysosomal suppression and decreased MyD88 signalling, HCQ may disrupt TLR7 and TLR9 ligand binding and TLR signalling in APCs, hence impeding TLR‐mediated activation of cells and cytokine generation. This medication also prevents the processing of antigens and subsequent MHC class II presentation to T cells in APCs, including pDCs and B cells. This stops the activation of T cell differentiation, thereby lowering the expression of co‐stimulatory molecules, like CD154, and it also lowers the amount of cytokines, like TNF, IL‐1 and IL‐6, that are produced through both T cells and B cells [9].

The pharmacological and/or side effects of chloroquine and HCQ are explained by a variety of mechanisms of action derived from in vitro research. Interestingly, it is yet unclear how these pathways relate to both safety and clinical effectiveness seen in vivo. Lysosomal action, autophagy and signalling networks are all directly impacted molecularly by antimalarial medications (Figure 2). There is additional information on how these medications affect B cells, T cells, additional antigen‐presenting cells and plasmacytoid dendritic cells (pDCs) (Figure 3). The process for action is likely dependent on context (i.e., reliant on the inflammatory conditions and/or damaged tissues or organs), as is the case with other immune system therapeutic approaches (Table 1) [9].

**TABLE 1:** Principal modes of activity of chloroquine and hydroxychloroquine.

	Mechanism
**1**	Inhibition of the synthesis of certain proinflammatory cytokines, including TNF, IFNα and IL‐1, that provides protection versus cartilage resorption caused by cytokines
**2**	Interference with the action of cyclic GMP‐AMP synthase (cGAS)
**3**	Inhibition of immunological stimulation, the presentation of antigen and MHC class II expression (lowering T cell expression of CD154)
**4**	Disruption of the signalling pathways for Toll‐like receptors 7 (TLR7) and 9

## 4 Combination of HCQ With Other Drugs in Therapy

The administration of chloroquine/HCQ was recently investigated when combined with various antiretroviral medications because it likely suppresses virus replication through an alternative process than that of presently prescribed antiretroviral medications. The discovery that chloroquine additionally displays anti‐HIV action in vitro against isolates from individuals experiencing failure of therapy with a multidrug‐resistant profile potentially supports the administration of chloroquine when combined with other antiretrovirals [10].

Recently conducted research has shown that leflunomide and HCQ together, at clinically relevant dosages, cumulatively suppress immunological stimulation, indicating that this medication combo may be used for the treatment of pSS [11].

## 5 HCQ'S Utilization for Rheumatic Disorders

**FIGURE 4:**
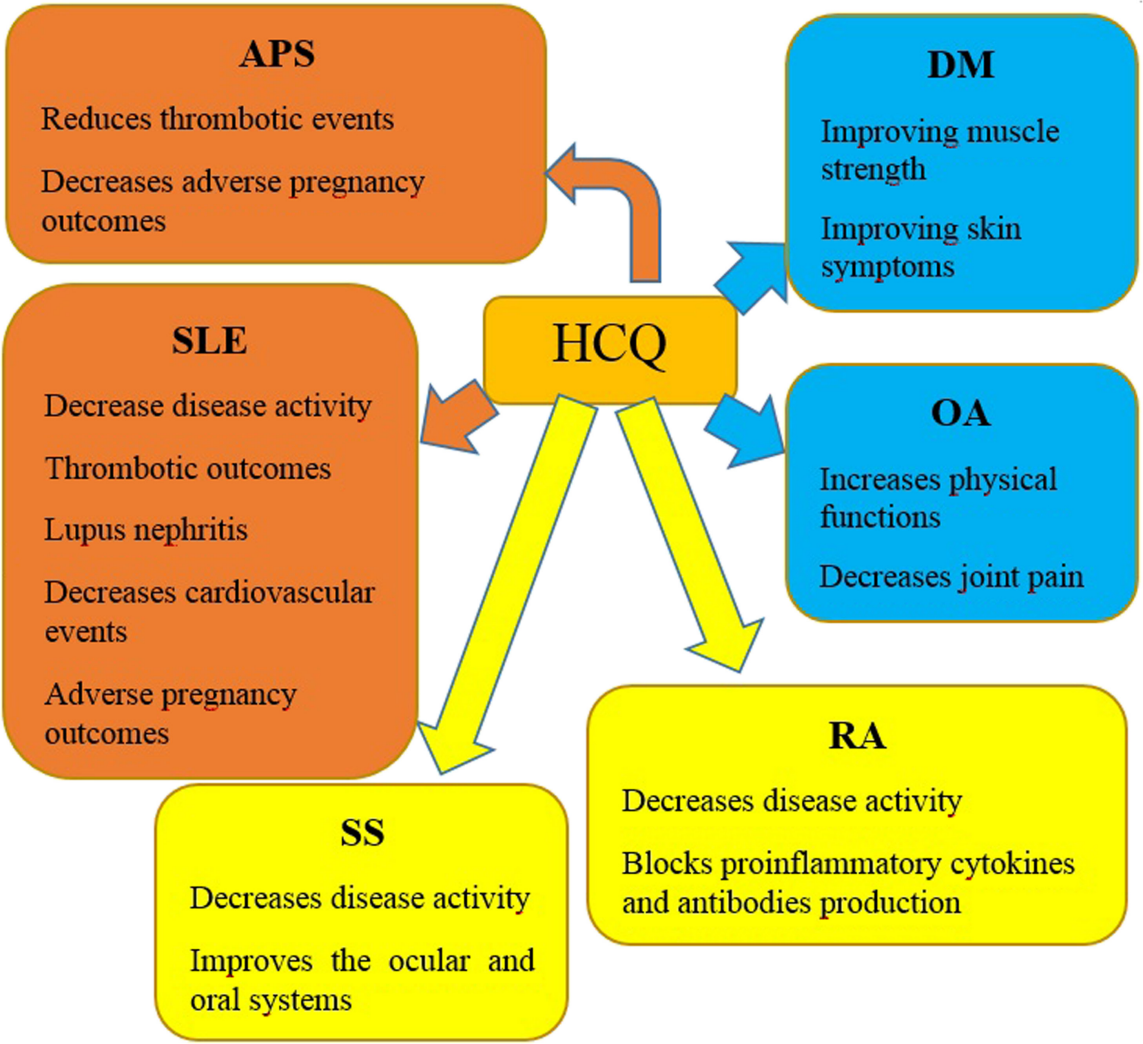
HCQ's utilization in rheumatism. The degree of proof confirming the administration of HCQ for each ailment is indicated by alternative hue coding. Orange indicates an elevated amount of proof, yellow indicates an average level and blue indicates a low level. APS (antiphospholipid syndrome); DM (dermatomyositis); HCQ (hydroxychloroquine); OA (osteoarthritis); RA (rheumatoid arthritis); SLE (systemic lupus erythematosus); SS (Sjögren's syndrome).

HCQ has shown significant benefits and may be employed to cure a variety of rheumatic conditions (Figure 4). The European League Against Rheumatism (EULAR) standards for various autoimmune illnesses were consulted in this evaluation in order to categorize the scientific evidence as low, medium or high [2].

## 6 Proof of Higher SLE and APS Levels

All individuals suffering from SLE should get HCQ medication, with an ideal daily dose of 5 mg/kg based on actual body mass, as recommended by EULAR. Earlier, HCQ therapy is advised to women with repeated premature deliveries, and HCQ has been suggested for APS attributable to SLE [12, 13].

## 7 Sjögren's Syndrome (SS) and RA as Indicators of Levels of Intermediate Severity

According to EULAR recommendations, methotrexate (MTX) is typically the recommended medication for RA. Leflunomide (LEF) + sulfasalazine (SSZ) + HCQ and classic trio treatment [MTX + SSZ + HCQ] are far better than monotherapy in situations with low efficacy, underscoring the significance of HCQ [14]. On the basis of moderate proof, HCQ is advised for the treatment of systemic disorders and the main triad of symptoms (pain, weariness and dryness) associated with SS [15].

## 8 Osteoarthritis (OA) and Dermatomyositis (DM) as Weak Evidence

According to EULAR recommendations, HCQ utilization is not advised for both of these scenarios, and the application of HCQ has only been documented in isolated investigations. HCQ use might be linked to a higher incidence of HCQ‐related rash, especially in people with diabetes mellitus [16].

## 9 HCQ and APS

Chronically positive antiphospholipid antibodies (aPL), abnormal pregnancies and arterial and/or venous thrombosis are the hallmarks of APS, an autoimmune illness. Adults who are young, primarily those within their ages of 15 and 50, are typically affected. With a male‐to‐female ratio of approximately 1:3.5 for primary APS and 1:7 for secondary APS, women are far more probable than men to have primary or secondary APS [17]. Less than 1% of all APS incidents are catastrophic APS, indicating its low frequency [18].

By preventing aPL‐stimulated immune cell induction, stimulation of platelets and hyperactivation of complements, HCQ has shown effectiveness in APS and can have an antithrombotic effect while lowering the frequencies of abnormal pregnancies. HCQ decreases dysfunction of endothelial cells, inflammation of the arteries and thrombosis in mice models of APS [19]. Regulations suggest using HCQ as an adjuvant treatment for APS‐related thrombosis in order to relieve thrombotic APS [20]. After 3 months of HCQ treatment for 22 aPL‐positive individuals, Schreiber et al. [21] observed a reduction in soluble tissue factor values. Other indicators of thrombotic propensity, including the activation of complement and annexin 5 exertion, did not, however, differ significantly. Nonetheless, HCQ can lower blood concentrations of C3a and C5a in APS individuals [22]. By blocking autophagosome‐lysosome fusion, HCQ may drastically decrease the proportion of NET‐positive neutrophils and NET release [23]. Furthermore, aggregation and activation of platelets are clearly inhibited by HCQ. In order to avoid and cure APS‐related thrombosis, NET inhibition decreases aggregation of platelets and drastically decreases circulating tissue factor concentrations [24].

More and more people are considering HCQ as an additional therapy for APS pregnancy. In women who have repeated pregnancy problems, the EULAR recommendations suggest raising heparin to therapeutic dosages, with HCQ taken into consideration throughout the preliminary trimester [25]. Alive rates of birth were 57% in the HCQ‐untreated subgroup and 67% in the HCQ‐treated subgroup in a retrospective cohort analysis of 170 pregnancies associated with 96 women, suggesting a correlation with greater rates of live birth [26]. The function of HCQ in individuals with APS or chronic aPL is presently being assessed in a multicentre trial that spans many European nations. Preterm birth (less than 34 weeks), insufficient placental development and early pregnancy loss are among the negative outcomes of pregnancy linked to aPL that the trial seeks to evaluate in relation to HCQ started prior to pregnancy and maintained for 9 months [27]. Despite taking therapeutic quantities of aspirin and enoxaparin prior to pregnancy, an individual with a record of catastrophic APS suffered developmental limitation in her sixth week of gestation; according to Mar et al. [28], intravenous immunoglobulin treatment and the addition of HCQ allowed the pregnancy to be successfully delivered to term. These results imply that HCQ may enhance pregnancy outcomes while avoiding catastrophic APS when used in conjunction with other medications.

## 10 HCQ and RA

At the start of 2016, it was believed that over 1.3 million people in the United States alone suffered from RA, a widespread ongoing autoimmune disorder that mostly impacts people between the ages of 20 and 50. It has been linked to higher mortality and a lower quality of life [29]. In order to manage the course of RA and attain eradication with reduced disease action, HCQ can be utilized as a supplement to DMARDs.

The pathophysiology of RA is linked to elevated levels of proinflammatory cytokines. The stimulating cytokines IL‐1, IL‐6, IL‐12, IL‐15, IL‐17, IL‐23 and B‐cell promoting factor are among those that HCQ can suppress and are implicated in the pathophysiology of RA. Additionally, it prevents the synthesis of autoantibodies and proinflammatory cytokines [7]. In a study of 325 individuals suffering from early RA, Schapink et al. [30] examined the effectiveness of HCQ‐MTX combination therapy in comparison to MTX monotherapy. Following 6 months, the MTX‐HCQ combined treatment group experienced a greater improvement in clinical manifestations and progression of the disorder. Individuals with RA benefit when HCQ is used to address cardiovascular issues associated with RA. Those receiving HCQ had a 72% lower rate of cardiovascular illnesses, according to a massive retrospective cohort research that lasted 12 years [31]. The probability of arrhythmias or ventricular arrhythmias is not increased by HCQ during therapy for RA, according to recent research [32]. Furthermore, better results for renal function have been linked to the usage of HCQ in individuals with RA. According to a previous extensive observational cohort research, individuals who had RA who received HCQ had a 36% lower risk of chronic renal disease than those who did not [33]. Both immediate and long‐term advantages are noted.

For the majority of individuals with RA, MTX is now thought to be the treatment of choice. Combination therapy with different medications is necessary because not all patients respond effectively to MTX monotherapy [34]. The recovery rate in the MTX + HCQ group was greater than in the MTX + LEF group (70.1% vs. 56.7%; *p* = 0.048) in a study comparing the effectiveness of RA patients receiving MTX + HCQ and MTX + LEF (97 patients in each group) [35]. Eleven and 16 months, respectively, were the median response times. Compared to individuals in the LEF group, more patients in the HCQ group experienced remission at the endpoint (46.8% vs. 32.5%; *p* = 0.063) and continued to have minimal activity of the disease (53.2% vs. 38.6%; *p* = 0.062). Additionally, glucocorticoid withdrawal was possible for many more individuals within the HCQ group (32% vs. 16.7%; *p* = 0.053). Further, the HCQ group saw an improvement in the increasing efficiency proportion [35]. The groups' levels of safety did not differ significantly [35]. In comparison to LEF, this suggests that HCQ combo treatment is more successful and cost‐efficient. Investigations evaluating activity of the disease along with other consequences in patients with RA acquiring MTX + HCQ + SSZ and those being given MTX + etanercept for 48 weeks revealed no substantial variations among the groups in terms of HCQ and biologics. These outcomes included pain, visualization progress, activity of the disease, health‐associated quality of life and essential drug‐associated incidents [36, 37]. Additionally, triple DMARD therapy was reported to be more cost‐effective than MTX + etanercept [37]. In contrast to conventional protocols comprising HCQ, a randomly assigned, unbiased investigator‐initiated study revealed a greater clinical rate of response at week 48 for abatacept and peficitinib versus those for tocilizumab [38]. Throughout the groups receiving treatment, radiographic progress was minimal and comparable [38]. In contrast to HCQ‐containing procedures, which are more cost‐effective, biologics are somewhat expensive, even though their therapeutic impact may be better [39]. Furthermore, inhibitors of Janus kinase (JAKi) are crucial in the management of those with active RA that is refractory. At Week 12, JAKi restores bodily activity, slows the advancement of imaging and alleviates RA manifestations and symptoms among individuals who have not responded well to MTX. Adalimumab has been shown to be more efficacious than JAKi [40]. While the inclusion of HCQ lowers the frequency of significant adverse events (such as severe infections and decreased liver function) and total adverse events, hence increasing survival, the administration of JAKi raises the possibility of becoming infected [41]. This suggests that when taken in combination with other medications, HCQ helps treat RA by increasing effectiveness and lowering adverse responses.

## 11 HQ and SLE

Numerous systems and organs are impacted by SLE, a persistent autoimmune illness with variable rates of incidence. According to a comprehensive review, the prevalence of SLE varied between 0.3 and 23.2 cases per 100 000 person‐years worldwide between 2013 and 2016 [42]. Young women among their ages of 15 and 45 are primarily affected. Consequently, SLE can be efficiently treated with HCQ. It builds up in lysosomes, where it raises pH levels to balance the environment that is acidic. This prevents class II major histocompatibility complex proteins from loading and presenting antigens. Furthermore, it partially disrupts the way that ribonucleic acid and deoxyribonucleic acid activate Toll‐like receptors [9].

In individuals with SLE, HCQ is linked to a decreased probability of thrombotic complications. In their comparison of 108 people suffering from SLE with no thrombosis and 54 individuals with prior thrombosis, Jung et al. [43] discovered that HCQ was linked to a reduced probability of thrombotic events. The researchers of a retrospective investigation of 1946 SLE individuals from Taiwan discovered that, over an average follow‐up duration of 7.4 years [hazard ratio, 0.91, 95% CI 0.71–1.15], patients with SLE who took HCQ during its initial year of therapy had a slightly lower probability of vascular events in comparison to those who did not [44]. HCQ has protective advantages as well. After using HCQ, individuals with inactive SLE had a 57% lower chance of experiencing serious illness action [45], and following the stopping of the medication, the clinical signs and illness behaviour worsened [46].

When it comes to treating individuals with SLE while pregnant as well as breastfeeding, HCQ offers benefits [47]. Individuals with SLE have a variety of autoantibodies, including anti‐Ro/SSA and anti‐La/SSB antibodies that are linked to congenital atrioventricular blockage and may penetrate the barrier of the placenta. Rates of recurrence can rise from 13% to 18%, especially if the mom has previous instances of fetal participation. In newborn hearts, HCQ can lower the prevalence of antibody engagement linked to SLE [48]. Furthermore, endometriosis is less likely to occur in SLE patients who continued being given HCQ medication [49].

In additional investigations, 826 individuals with SLE who were receiving HCQ were included. 795 patients were still included in the trial after more than a year of monitoring [50]. Continuous HCQ administration was linked to a lower risk of coronary artery disease in sufferers of SLE who utilized HCQ for at least 318 days, even after controlling for chronic comorbidities. In addition to protecting the heart, HCQ also decreased the probability of coronary artery disease [50, 51]. Arrhythmias and ventricular arrhythmias were not made more likely by it [32, 52]. HCQ lowers the individual's possibility of developing chronic renal disease in combination with cardiovascular illness [53].

According to an investigation done in the United States with 30 086 patients with SLE, HCQ was the drug most often prescribed medication [54]. Despite its effectiveness, corticosteroids have side effects including infections and elevated levels of blood pressure. When HCQ is added to immunosuppressants (like mycophenolate, tacrolimus, cyclosporine, MTX and azathioprine) in those suffering from SLE, irrespective of prior therapy, it not only lowers the activity of the disease, but also permits a steady decrease of corticosteroid dosages [55], which lowers the risk of adverse reactions.

## 12 HCQ and SS

A lifelong and systemic autoimmune illness, PSS is thought to impact 0.06% of people globally [56]. It is marked by localized lymphocytic infiltration of the glands that produce saliva, which causes pain, exhaustion and dry mouth and eyes. These signs, which impact over 80% of individuals, can have a major impact on their lives and careers [57]. At the level of the cells, HCQ inhibits autophagy, which stops multiple kinds of cells from being immunely activated. This blockade might represent an essential process in the therapy of SS since it lowers the production of cytokines and controls CD154 expression in T cells [9].

Ocular signs are greatly reduced and systemic harm is avoided when HCQ is used for the management of pSS [58, 59]. Nevertheless, a randomized study showed that during the course of 24 weeks of medication, HCQ did not significantly ameliorate signs for individuals with pSS in comparison to a placebo [60]. According to a meta‐analysis conducted by Wang et al. [61], there were additionally no appreciable variations in eye dryness or mouth dryness among the HCQ‐treated and placebo groups in pSS individuals. According to a previous meta‐analysis, HCQ dramatically reduced erythrocyte sedimentation percentage, C‐reactive protein and IgM and IgA levels, as well as other associated measurements and oral symptoms. Nevertheless, there was no discernible improvement in other clinical characteristics, such as ocular engagement, fatigue, joint inflammation, signs of neurological disease, pulmonary symptoms, lymphoid hyperplasia, renal failure and parameters of experimentation [62]. Arrhythmias or ventricular arrhythmias are not made more likely by HCQ therapy for SS [32]. There are at present no medications that can treat pSS. It is still critical to reduce symptoms and avoid problems. For the management of pSS, rheumatologists must thus develop and find viable immunosuppressive medicinal drugs.

## 13 HCQ Along With Various Rheumatic Conditions

HCQ appears to alleviate DM skin symptoms and decrease the need for corticosteroids for managing DM inflammation of the skin [63]. Individuals with diabetes mellitus benefit from HCQ for both systemic and cutaneous symptoms. After 3 months of HCQ therapy, nine participants with youth‐onset DM who failed to respond well to prior systemic corticosteroid therapies demonstrated better skin rashes and notable gains in abdominal and proximal muscular strength, according to a retrospective series of cases [64]. HCQ, nevertheless, may exacerbate inflamed skin symptoms in young people with diabetes mellitus [65].

Within the conclusion of 24 weeks, the HCQ group (200 mg twice daily) in a controlled, randomized study of knee OA demonstrated enhanced bodily activity and less knee discomfort than the placebo group [66]. But the most recent meta‐analysis found that in comparison to a placebo, HCQ showed a minor effect on decreasing functioning and a minor and not statistically significant influence on lessening knee and hand OA discomfort. Regarding hand OA, there was no apparent enhancement in the standard of life [67]. Consequently, depending on their individual circumstances, people suffering from DM and OA should receive HCQ.

## 14 Serious HCQ‐Related Adverse Effects

HCQ is thought to act as immunomodulatory instead of immunosuppressive medications, and they have a well‐established and favourable safe record. HCQ treatment is not linked to a higher risk of cancer [68] or infectious problems [69]. Certain antimalarial medications most frequently cause adverse reactions in the gastrointestinal tract, involving nausea, vomiting, diarrhoea and abdominal pain [70]. Moreover, a number of investigations have documented the appearance of cardiomyopathy among individuals with rheumatic illnesses [71] as well as HCQ‐associated myopathy [72] and HCQ‐mediated and/or chloroquine‐mediated cardiotoxic effects, such as rhythm abnormalities (like a prolonged QT interval). The bioavailability of antimalarial medications and the likelihood of side effects can both be increased by kidney failure.

Considering the suggested HCQ dosages, there has been a bit of disagreement in the available literature. As long as the HCQ dose is adjusted to the proper weight for height, a dosage of ≤ 6.5 mg/kg of optimum body mass is generally regarded as harmless [73]. Clinical studies that show that HCQ is frequently prescribed excessively in obese people due to the minimal retention of HCQ in fat tissue, thereby increasing the likelihood of adverse effects, served as the basis for this opinion [73, 74]. The preferred dose has been changed to ≤ 5 mg/kg over the past few years depending on the participants' exact body mass [74]. Continuous (often defined as > 1 year) usage of HCQ has an increased chance of adverse effects, which are mostly influenced by the amount taken every day in relation to the patient's weight [75]. The suggested HCQ dose has been reduced from 6.5 mg/kg daily to 5 mg/kg by US and UK standards [76].

**FIGURE 5:**
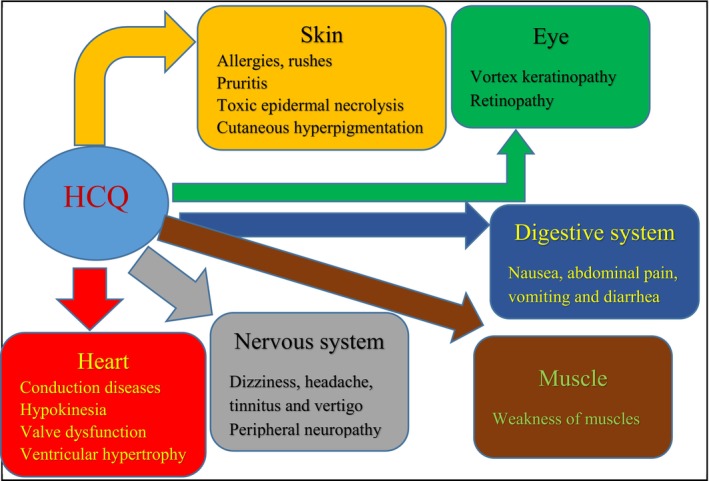
Adverse HCQ‐related effects.

Despite being safe, tolerated well and typically efficient, HCQ has been known to cause certain negative side effects (Figure 5). Gastrointestinal problems, including diarrhoea, vomiting, nausea and abdominal discomfort, are among the most frequent side effects. These issues might be connected to changes in the microbiota brought on by HCQ [47].

## 15 Neurotoxic Effects

Headache, vertigo, dizziness and tinnitus are examples of toxicities of the central nervous system. There have been some documented occurrences of epilepsy linked to psychosis and a lowered seizure threshold, particularly when HCQ and cortisol are taken together [77]. Perineural and Schwann cell injury seem to be linked to damage to nerves [78]. Via nerve biopsies, Pagès and Pagès [79] discovered demyelination linked to inclusions of cytoplasm within Schwann cells. Pseudo‐Parkinsonism has been observed infrequently, as has neurotoxicity [80]. The importance of regular evaluation for long‐term neuromuscular toxicity has not been shown by clinical trials. To find out if there is a transmission disease, it is advised that the peripheral nerves be thoroughly inspected employing electromyography if a patient shows signs of nervous system abnormalities. EEG, MRI or brain CT can also be used for ruling out anomalies of the central nervous system.

## 16 Dermal Toxic Effects

**FIGURE 6:**
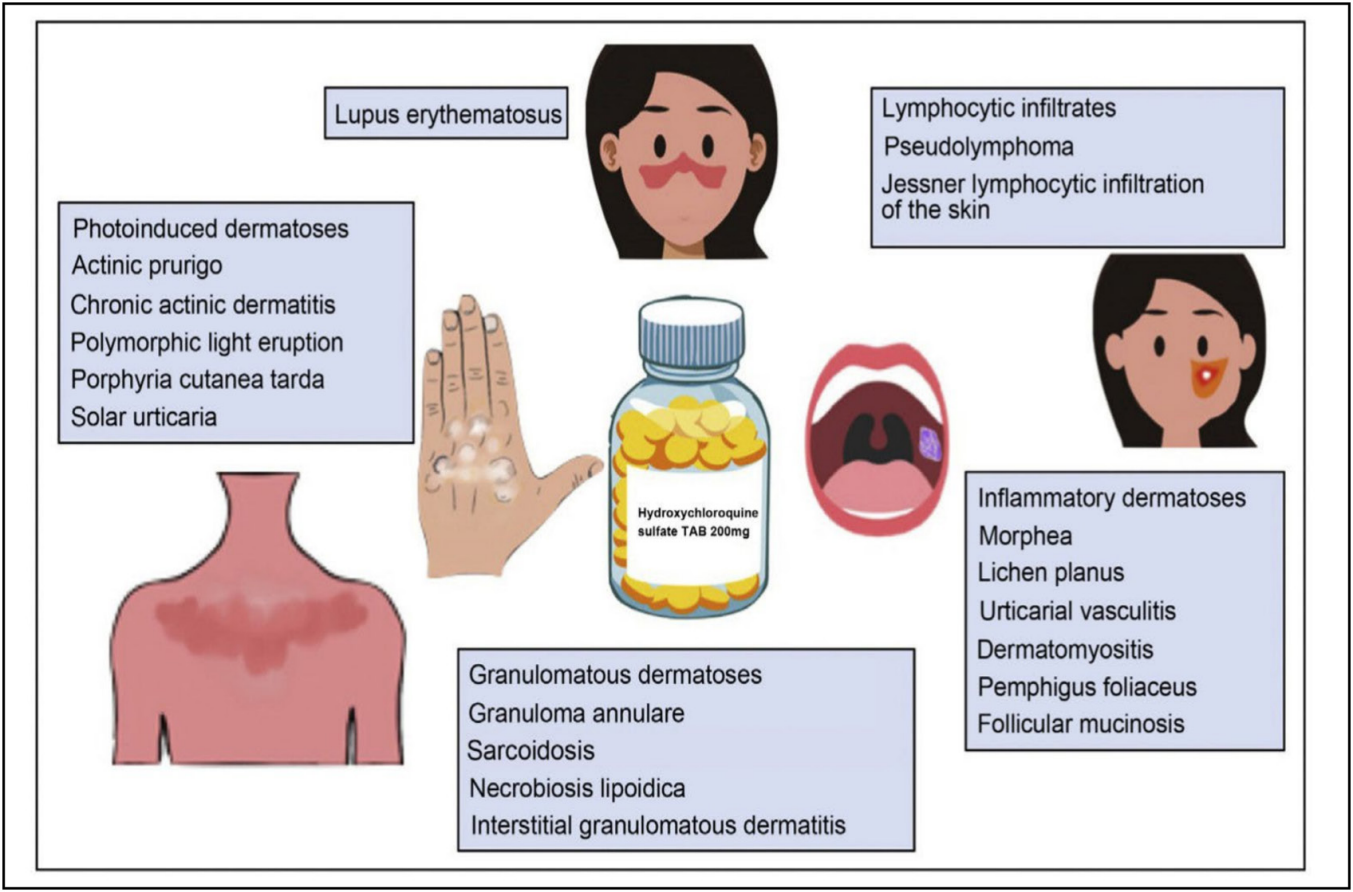
Hydroxychloroquine in dermatology [83].

It is frequently used without a prescription in a variety of autoimmune and inflammation‐related skin disorders and is a primary remedy for lupus erythematosus in dermatology (Figure 6). Utilizing HCQ may result in negative dermatological consequences that range in seriousness. Allergies or nonspecific causes might result in acute dermal responses, including rashes or medication eruptions [81]. Prolonged use may result in skin and oral mucosal hyperpigmentation and pruritus. It is anticipated that less than 10% of patients experience HCQ‐stimulated pruritus, while 10%–20% of patients experience hyperpigmentation [82].

According to 94 publications in a comprehensive analysis of HCQ's dermatological side effects, individuals who had SLE (72%) or RA (14%) participated in the majority of cases, respectively. The cumulative dosages that varied from 3 to 2500 g were associated with adverse cutaneous responses. Alopecia (12 cases), stomatitis (11 cases), generalized acute exanthematous pustulosis (27 cases), Stevens‐Johnson syndrome or toxic epidermal necrolysis (26 cases), hyperpigmentation (116 cases), pruritus (62 cases) and medication eruptions or rashes (358 cases) were the most frequent dermatological adverse effects [84]. Particularly, Black individuals are more likely to experience acute pruritus and cutaneous depigmentation, which exhibit preferences for races [85]. When HCQ is stopped, pruritus usually goes away fully, while hyperpigmentation could only go away partly [86]. In the event of an allergic response, the medication should be stopped right away. With HCQ treatment, routine skin examinations, such as dermoscopy and skin biopsies if required, are advised to check for dermatological issues.

## 17 Toxic Effects Related With Muscles

Elevated creatine kinase concentrations and corresponding histological characteristics are indicators of muscle toxicity linked to HCQ, according to investigations [87, 88]. The proximal weakness of the muscles with no myalgia or increased enzyme activity is a common symptom of myopathy; in serious situations, breathing difficulties may ensue [87]. After stopping the medication, these problems frequently get better [89]. Myositis, myasthenia and weakness in the limbs are uncommon symptoms of muscular poisoning [80].

**FIGURE 7:**
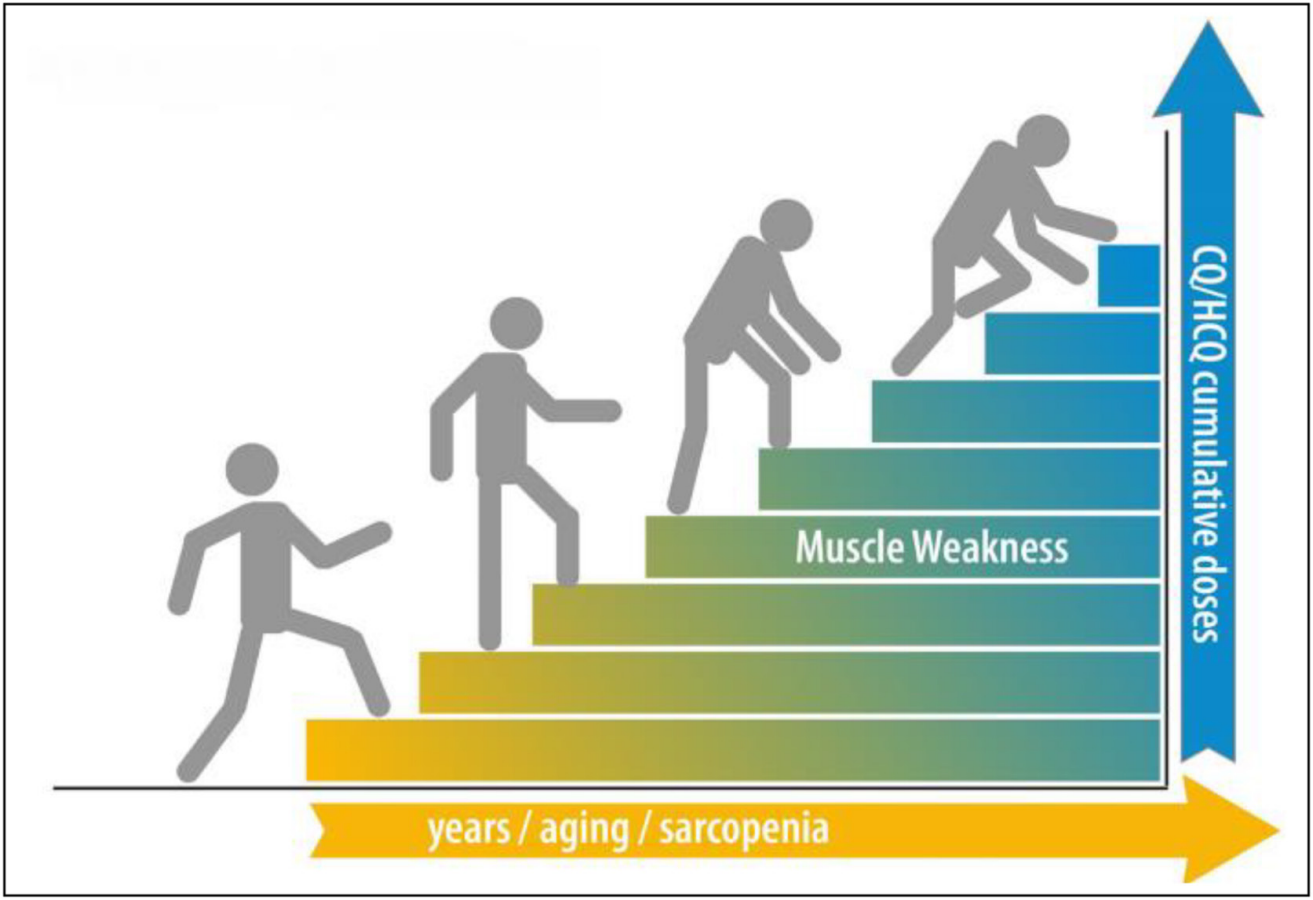
Hypothesis for combined effects of sarcopenia and CQ/HCQ‐induced myopathy, along with aging and chronic use of drugs [90].

As a hypothesis to be investigated (regression analysis correlating age with patient symptoms), sarcopenia may be more than a confounding factor of drug‐induced muscle weakness in these patients, but a co‐factor affecting the overall quality of the muscle and causing a decrease in muscle strength/power in these populations [90] (Figure 7). It is advised to employ muscle biopsy and electromyography to verify the identification of HCQ‐stimulated myopathy if muscular toxicity is thought to occur during HCQ use. The concentrations of muscle enzymes should also be dynamically monitored.

## 18 Ocular Toxic Effects

**FIGURE 8:**
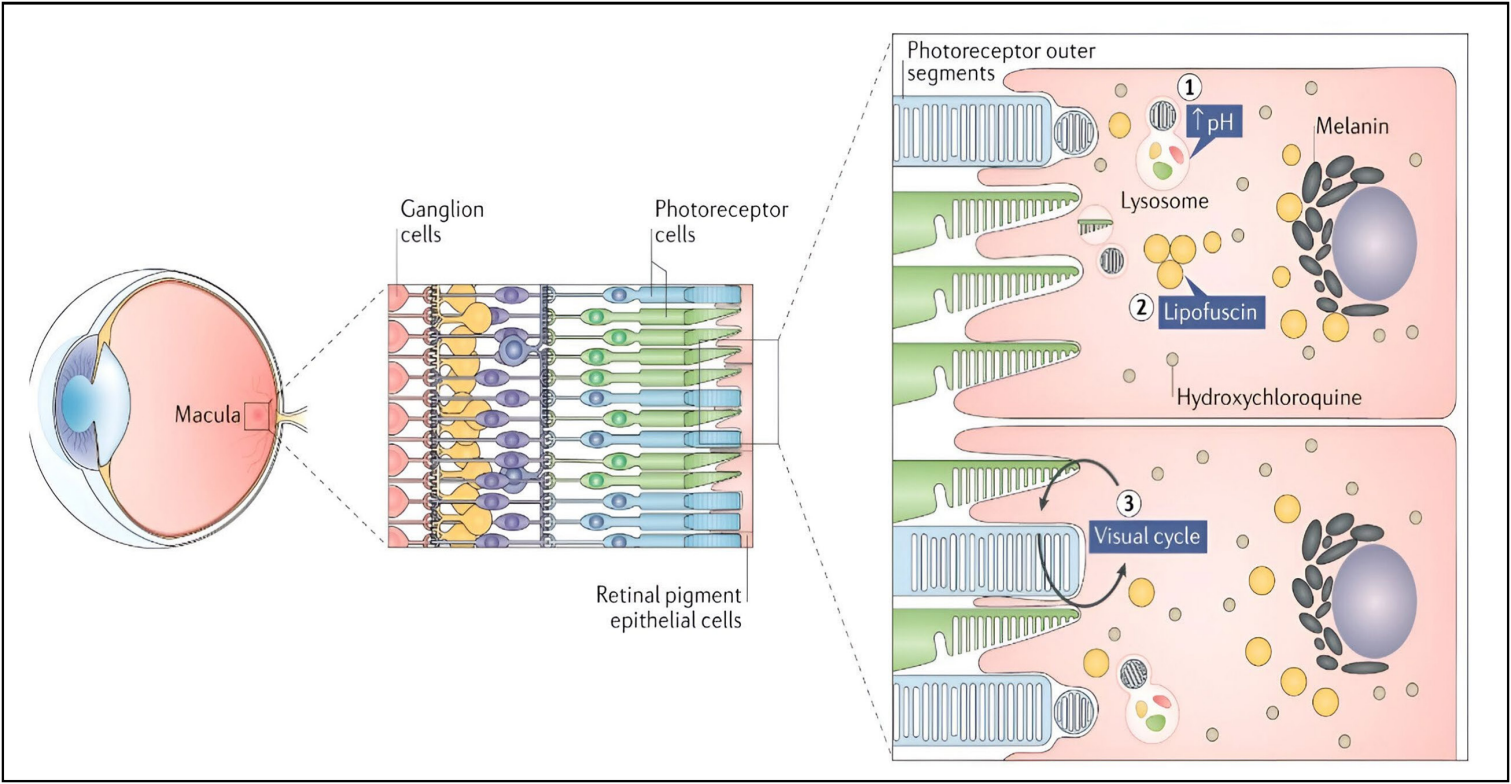
Mechanisms of hydroxychloroquine retinopathy [89].

Retinopathy can result from HCQ build‐up in the eye, which harms photoreceptor cells in the epithelium of the retinal pigment and causes progressive perifoveal degeneration [91]. The length of therapy is linked with this toxicity. At the suggested dosages, the 5‐, 10‐ and 20‐year toxicity probabilities for retinopathy have been determined to be less than 1%, 2% and 20%, respectively. After 20 years of use, a person's risk of toxicity rises by 4% every year [92]. The toxicity risk elevates five to seven times after daily intake of HCQ surpasses 5 mg/kg/d [93]. Annular black spots surrounding the fovea are the first sign of this poisoning [94], and they can expand over time and cause blindness or severe vision loss. Bull's‐eye maculopathy, a ring of retinal loss of pigment in the parafoveal area, is generally indicative of late‐stage HCQ retinopathy [95]. Drug accumulation in the retinal pigment epithelial might explain why hydroxylchloroquine retinopathy continues to progress after drug cessation in patients [96] (Figure 8).

Once toxicity is identified, HCQ must frequently be stopped in clinical practice. Nevertheless, severe or moderate retinopathy may continue to cause retinal harm despite stopping treatment, perhaps as a result of previous injury to retinal pigment epithelial cells that resulted in the demise of photoreceptors [97]. It is advised to do baseline retinal exams prior to starting HCQ treatment. In the lack of significant risk factors, annual screening ought to start 5 years following treatment. The yearly screening should begin sooner if there are risk factors, including older age or liver disease. Automatic field of vision evaluation with spectral‐domain optical coherence tomography is a suggested test. Other tests, including multifocal electroretinography, that offer unbiased data on the field of view, may be necessary in specific situations, especially for Asian subjects [98].

## 19 Cardiovascular System Toxicity

**FIGURE 9:**
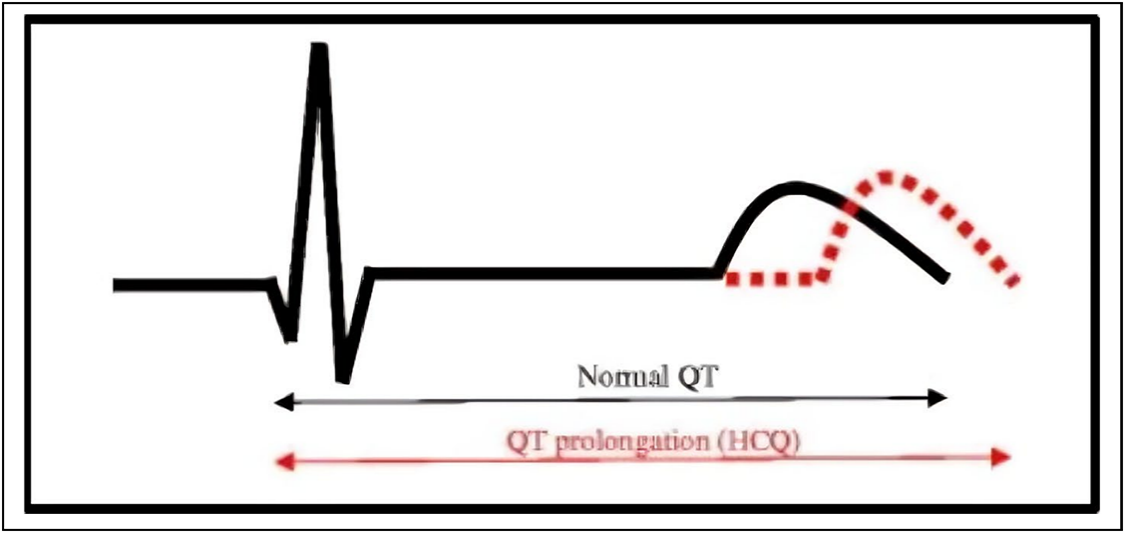
Prolonged QT periods due to HCQ treatment [100].

A major adverse effect is cardiac toxicity. Long‐term QT periods, increased biomarkers of cardiac activity, sick sinus syndrome, transmission abnormalities and cardiac failure can all result from long‐term HCQ administration [84, 86, 99, 100] (Figure 9).

The majority of the 127 patients in the systematic study had either RA (*n* = 28) or SLE (*n* = 49). HCQ was administered to 39.4% of those individuals. Given an average overall dosage of 803 g and a greater cumulative amount of 1235 g, the majority of patients received treatment for a longer duration of time (with a median 7 years, ranging 3 days to 35 years). For 85% of the individuals, the primary adverse impact was conductivity abnormalities. The ventricular hypertrophy, hypokinesia, failure of the heart, hypertension of the pulmonary arteries and valve malfunction were among the other nonspecific adverse cardiac events [101]. Individuals who show symptoms of myocardial toxicity are screened for cardiovascular biomarkers, such as troponin and brain natriuretic peptide, and undergo cardiovascular MRI, and endomyocardial biopsy in order to diagnose HCQ toxicity and direct therapy [60]. Upon beginning HCQ treatment, individuals who have autoimmune illnesses should have cardiac screening. To completely rule out disorders related to the heart, routine procedures like cardiovascular ultrasounds and cardiac electrocardiograms are conducted. It is not advised to use HCQ if a significant conduction obstruction is identified. It is advised to cease taking HCQ right away if heart illness appears while receiving treatment. But after stopping, only 45% of those treated totally recovered [96]. Further cardiac examinations and, if required, the prescription of suitable drugs should be carried out if the problem fails to get better [102]. As a result, heart screening is crucial for individuals with rheumatic diseases receiving HCQ.

## 20 HCQ and COVID‐19

The WHO formally designated the virus that sparked a worldwide pandemic as COVID‐19 in February 2020 [103]. Apart from antiviral medications like lopinavir/ritonavir and remdesivir, HCQ also prevents the growth of viruses [104]. Through raising intracellular endosomes' pH, HCQ has been demonstrated in vitro to prevent viral entrance, replication and glycosylation of the surface of the viral receptor angiotensin‐converting enzyme 2 [105]. Inflammation is the primary feature in the initial phases of COVID‐19, and in serious instances, cytokine storms can develop, which would be detrimental to the patient prognosis [106]. HCQ may help those who have COVID‐19 because of its anti‐inflammatory and immunomodulatory qualities in addition to its capacity to control proinflammatory cytokines like TNF, IL‐1 and IL‐6 [107].

Azithromycin (500 mg on Day 1 and 250 mg daily for the following 4 days) and HCQ (600 mg/d for 10 days) were administered to 80 COVID‐19 patients in an earlier trial. In 83% of individuals, nothing infectious was found in their nasopharyngeal specimens on Day 7. By Day 5, viral culture results were negative in 97.5% of respiratory samples [108]. These findings imply that HCQ might have antiviral properties. There was nothing compared with HCQ monotherapy in this trial, though.

Participants were split into two different groups according to their therapy choices in a prospective randomized cohort investigation: 56 were placed in the tocilizumab‐HCQ group and 52 in the tocilizumab‐remdesivir group. Following therapy, every group showed a considerable rise in the PaO2/FiO2 ratio and a substantial decrease in C‐reactive protein [109]. The tocilizumab‐HCQ subgroup showed a substantial reduction in low‐density lipoprotein, ferritin and D‐dimer levels [109]. Myocarditis (15.4%), pulmonary embolism (7.7%) and secondary bacterial infections (42.3%) were among the adverse reactions that only happened after tocilizumab administration [109]. These results imply that HCQ may be an efficient and secure therapy for COVID‐19 when taken in combination with other medications. Conversely, the addition of HCQ to conventional therapy resulted in a significant decline in clinical function, an increased probability of kidney failure and an increased need for invasive mechanical ventilation among individuals with severe COVID‐19 [110]. When contrasted with conventional therapy, HCQ, either by itself or in conjunction with azithromycin, failed to enhance the clinical situation over a 15‐day period in patients diagnosed with mild‐to‐moderate COVID‐19. It was also linked to higher levels of liver enzymes and longer QT periods [111]. In the end, a number of variables lead to contradictory findings, such as small numbers of samples, the absence of randomized and placebo‐controlled trials, single‐center designs, low‐quality methods, different initial characteristics and potential biases, even though certain small studies indicate that HCQ is linked to shorter recovery times in COVID‐19 [112]. There is still little and conflicting data on how HCQ affects viral load, illness development, death from all causes and symptom relief [113]. Some individuals may experience haematological problems and cardiotoxicity (including QT prolongation, ventricular arrhythmias and cardiac arrest) as a result of the extensive use of HCQ [114]. As a result, individuals should be continuously followed during medication, and HCQ ought to only be administered for COVID‐19 in compliance with national, regional or local treatment recommendations.

As a conclusion, additional research on the cellular and molecular pathways of HCQ has demonstrated that it regulates cell‐mediated reactions and molecular mechanisms resulting in an immunomodulatory effect. The inhibition of inflammatory reactions, either directly or indirectly, has several consequences. HCQ is frequently prescribed for managing rheumatic conditions and greatly enhances patients' standard of life. HCQ is generally regarded as secure. Even though adverse effects are rare, they can nonetheless happen and have a detrimental impact on patients' lives. The majority of side effects are linked to long‐term use and significant dosage accumulation. Throughout HCQ treatment, it is crucial to keep an eye on pertinent signs, perform the necessary tests and handle adverse responses as soon as possible.

## Author Contributions

Ilker Ates and Hilal Sahin handled the planning, search strategy, initial draft authoring and data extraction. Lalu Muhammad Irham, Sinan Suzen and Serkan Yilmaz edited, revised and prepared the final document.

## Conflicts of Interest

The authors declare no conflicts of interest.

## Data Availability

No novel information was generated or analysed in the development of this article. The information obtainable from the cited sources was utilized if appropriate.
